# *Mycobacterium tuberculosis* Beijing Genotype, Northern Malawi

**DOI:** 10.3201/eid1101.040869

**Published:** 2005-01

**Authors:** Judith R. Glynn, Amelia C. Crampin, Hamidou Traore, Malcolm D. Yates, Frank D. Mwaungulu, Bagrey M. Ngwira, Steven D. Chaguluka, Donex T. Mwafulirwa, Sian Floyd, Caroline Murphy, Francis A. Drobniewski, Paul E.M. Fine

**Affiliations:** * London School of Hygiene and Tropical Medicine, London, United Kingdom; † Karonga Prevention Study, Chilumba, Malawi; ‡ Kings College Hospital (Dulwich), London, United Kingdom

**Keywords:** tuberculosis, RFLP, Africa, drug resistance, dispatch

## Abstract

In a 7-year population-based study in Malawi, we showed that Beijing genotype tuberculosis (TB) increased as a proportion of TB cases. All the Beijing genotype strains were fully drug sensitive. Contact histories, TB type, and case-fatality rates were similar for Beijing and non-Beijing genotype TB.

The Beijing genotype family of *Mycobacterium tuberculosis* may be spreading worldwide. The genotype may be particularly virulent and have a predilection for developing drug resistance; the multidrug-resistant W strain is a member of this family (*1*). Few studies have examined trends in the prevalence of Beijing genotype strains over time, and little is known about their distribution or characteristics in Africa (*2*), although drug-resistant Beijing genotype strains have been identified in Cape Town and Nairobi (*3*,*4*). We describe Beijing genotype tuberculosis (TB) in a population-based, 7-year study in Malawi.

## The Study

As part of the Karonga Prevention Study, a longstanding epidemiologic study of TB and HIV in a rural area of northern Malawi (*5*,*6*), persons suspected of having TB in Karonga District are identified at the hospital and peripheral clinics. Sputum is taken for smear and culture. Cultures are also performed on lymph node aspirates and other samples as appropriate. Treatment follows Malawi National TB Control Programme guidelines, and drug resistance has remained low, with initial isoniazid resistance ≈6% during this study (*7*,*8*).

Positive cultures from all patients in whom TB has been diagnosed since late 1995 have been DNA fingerprinted (*9*). Fingerprinting used IS*6110* restriction fragment length polymorphism (RFLP) following standard guidelines (*10*), and RFLP patterns were compared by using computer-assisted (Gelcompar 4.1, Applied Maths, Kortrijk, Belgium) visual comparison. Beijing genotype strains were identified by comparing each RFLP pattern to 19 reference RFLP patterns (https://hypocrates.rivm.nl/bnwww/index.html) with 1% position tolerance. Taking strains with patterns with >80% similarity to any of the reference patterns as Beijing genotype, and spoligotyping strains showing 75%–80% similarity, this method has been shown to identify Beijing genotype strains with a sensitivity of 96% to 100% and specificity of 98% to 100%, using spoligotyping as the gold standard (*11*). In the present study, we spoligotyped all strains with RFLP patterns with >70% similarity to the reference patterns in order to maximize the sensitivity and specificity for identifying Beijing genotype strains.

From late 1995 to early 2003, a total of 1,194 specimens were fingerprinted from 1,044 persons (84% of patients in whom culture-positive TB was diagnosed in this period). After excluding likely laboratory errors (*9*) (25 isolates) and multiple isolates per person, we found 406 different RFLP patterns in samples from 1,029 patients. Overall, 757 patients (73.6%) were clustered, i.e., they had an isolate with an RFLP pattern that was identical to that of at least 1 other patient in the study.

**Figure 1:**
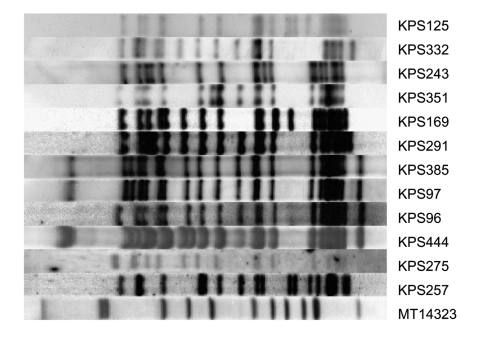
Restriction fragment length polymorphism patterns of 12 Beijing strains from Karonga District, Malawi. All strains were >79% related to at least 1 of the other Beijing strains found in the district. Strain MT14323 is a reference strain.

Thirteen different RFLP patterns from isolates from 45 patients matched the Beijing genotype reference patterns by >75%. At least 1 isolate from each of the 13 strains was spoligotyped: 12 patterns were confirmed as Beijing genotype. The RFLP pattern from the strain with the non-Beijing genotype on spoligotyping, present in only 1 patient, matched the RFLP pattern of a reference strain by 81% but showed a <73% match with the other reference RFLP patterns and a <71% match with the other 12 Beijing genotype RFLP patterns from Malawi. In the whole dataset, only 3 other strains had RFLP patterns that matched the reference set by >70% (and <75%): spoligotyping confirmed that these strains were not Beijing genotype. The RFLP patterns of the 12 Beijing genotype strains from Karonga District are shown in Figure 1.

Overall 44 (4.3%) of 1,029 patients had Beijing genotype TB (Table). Beijing genotype strains were found in persons from all areas of the district, and they were no more common in those who had lived outside Malawi. Of the 8 people with Beijing genotype TB who had been born outside Malawi or lived outside Malawi in the last 5 years, 4 were from Tanzania and 4 from Zambia. Among TB patients, no association was seen between Beijing genotype and HIV status. Beijing genotype strains were more common in women than in men, and this difference persisted after adjusting for age and year of diagnosis (adjusted odds ratio [OR] 2.9, 95% confidence interval [CI] 1.4–6.0).

**Table:** Comparison of persons with Beijing and non-Beijing genotype *Mycobacterium tuberculosis*

Characteristic	Beijing genotype, n (%)	Other genotypes, n (%)	p value
Sex			0.001
Male	10 (22.7)	472 (47.9)	
Female	34 (77.3)	513 (52.1)	
Age group			0.3
<15	2 (4.6)	16 (1.6)	
15–29	17 (38.6)	306 (31.1)	
30–44	16 (36.4)	423 (42.9)	
>45	9 (20.5)	240 (24.4)	
Born in Malawi			0.08
Yes	38 (90.5)	747 (79.2)	
No	4 (9.5)	196 (20.8)	
Moved in last 5 y			0.8
No move	16 (40.0)	343 (41.2)	
Within district	10 (25.0)	188 (22.6)	
Outside district	9 (22.5)	226 (27.1)	
Outside country	5 (12.5)	76 (9.1)	
HIV status			0.4
Positive	15 (57.7)	396 (65.6)	
Negative	11 (42.3)	208 (34.4)	
Previous tuberculosis (TB)			0.9
Yes	3 (6.8)	74 (7.6)	
No	41 (93.2)	904 (92.4)	
BCG scar			0.7
Yes	23 (74.2)	486 (69.7)	
No	6 (19.4)	176 (25.3)	
Doubtful	2 (6.5)	35 (5.0)	
Contact with TB patient in district			0.6
Yes	22 (50.0)	453 (46.0)	
No	22 (50.0)	532 (54.0)	
Type of TB			0.4
Smear-positive pulmonary	33 (75.0)	711 (72.2)	
Smear-negative pulmonary	10 (22.7)	202 (20.5)	
Extrapulmonary	1 (2.3)	72 (7.3)	
Drug resistance			0.2
Sensitive	44 (100)	920 (93.9)	
Resistant isoniazid only	0	38 (3.9)	
Resistant isoniazid plus	0	22 (2.2)	
Died during treatment			0.7
Yes	13 (32.5)	224 (29.6)	
No	27 (67.5)	532 (70.4)	

**Figure 2:**
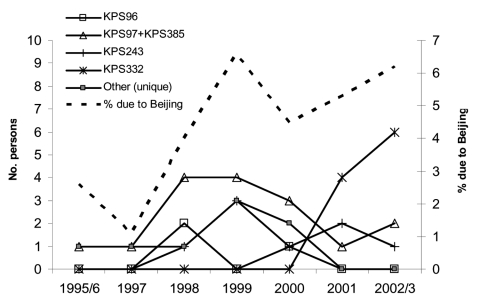
Beijing genotype tuberculosis (TB) in Karonga District, Malawi, over time. The solid lines show the number of persons with each Beijing genotype restriction fragment length polymorphism (RFLP) pattern, and the dotted line shows the proportion of culture-positive TB cases that are due to the Beijing genotype. Because strains KPS97 (14 patients) and KPS385 (2 patients) differed by only 1 band on RFLP, they are shown together.

The proportion of TB due to Beijing genotype strains increased over time (Figure 2, OR 1.2, 95% CI 1.0–1.4 for each year of study, p = 0.03) and was slightly lower in older persons (p = 0.2, test for trend). Of the 12 different Beijing genotype RFLP patterns, 5 were shared by >2 patients (i.e., were clustered), and the remaining 7 were unique in the dataset. The proportion of patients who were clustered was slightly higher in those with Beijing genotype strains than among those with other strains (84% vs. 71%, p = 0.07, after excluding those with <5 bands on the RFLP pattern).

Both the time trend and the association with age suggest an increase in Beijing genotype TB, and the association with clustering might suggest higher transmissibility. These results were influenced by the appearance of strain KPS332 from October 2001 onwards, affecting 10 patients. This strain was 83% similar to strain KPS96 and more distantly related to the other strains, so this strain was probably newly introduced into the district. However, an increase over time was also apparent in the data before 2001 (OR 1.3, 95% CI 0.98–1.8, for each year of study, p = 0.07), and the trend with age was stronger for this earlier period (p = 0.07).

Since 1997, patients have been asked about contacts they had had with persons in whom TB had been diagnosed. Further potential source contacts were identified from the project database by identifying relatives and those sharing a household. The diagnosis of TB in these named contacts was checked with the project database (*9*). Two patients with strain KPS332 were relatives.

Overall, approximately half of those with Beijing genotype TB and of those with other strains had identified contact with previous TB patients (Table). Among the 219 persons who were named as potential source contacts and had RFLP results, 10 (4.6%) had Beijing genotype strains, similar to the proportion in the whole population. Some contacts were named more than once. The proportion of case-patients who had identical RFLP patterns to these source contacts, confirming transmission, was similar for contacts with Beijing genotype strains (3 [20%] of 15 contact pairs) and those with other strains (61 [21%] of 289, p = 0.9). Since the proportion of contacts with the Beijing genotype is similar to the proportion with Beijing genotype in the whole population, and since the proportion of transmissions that were confirmed by RFLP was similar for those with Beijing genotype and those with other genotypes, this analysis provides no evidence of increased transmissibility of Beijing genotype strains. In addition, those with Beijing genotype were no more likely to be smear-positive than were those with other genotypes (Table).

Drug resistance was tested to isoniazid and rifampicin (rifampin). If resistance was found, sensitivity to other drugs was tested as well. None of the Beijing genotype strains had any drug resistance, compared to 6.1% of other strains (p = 0.1).

Outcome data were available for 92% of patients. After excluding 154 patients (3 with Beijing genotype) who were lost to follow-up or transferred, we found that the proportion who died during treatment was similar for those with Beijing genotype strains as for the other patients (Table). These results were little altered by adjusting for year of diagnosis, age, sex, or HIV status.

## Conclusions

Beijing genotype strains of *M. tuberculosis* are present in northern Malawi. The variety of RFLP patterns suggests several different introductions of Beijing genotype strains into the district. The origin of these strains is unknown, but they may have come directly from Asia, where they are common (*12*): Chinese people have worked in the district, for example, as agricultural advisors, in the last 20 years. The proportion of TB cases caused by Beijing genotype strains increased over the period of the study. This increase was exacerbated by 10 cases with a single RFLP pattern occurring within the last 12 months, which suggests an outbreak, although epidemiologic links were only established for 2 cases. No association with drug resistance was seen, and no evidence of increased severity or transmissibility of TB was seen in those with Beijing genotype.
